# The protective effects of S14G-humanin (HNG) against lipopolysaccharide (LPS)- induced inflammatory response in human dental pulp cells (hDPCs) mediated by the TLR4/MyD88/NF-κB pathway

**DOI:** 10.1080/21655979.2021.1979914

**Published:** 2021-10-04

**Authors:** Ping Zhang, Zhiqiang Cui, Shuai Li

**Affiliations:** Department of Stomatology, Heji Hospital Affiliated of Changzhi Medical College, Changzhi, Shanxi, China

**Keywords:** Pulpitis, S14G-humanin, dental pulp cells, NF-κB

## Abstract

Pulpitis is reported in large populations of patients and significantly impacts their normal life quality. It is reported that the lipopolysaccharide (LPS) in Gram-negative bacteria induces severe inflammation in dental pulp tissues. S14G-humanin is a derivative of humanin and has been recently confirmed to possess promising anti-inflammatory properties. The current study aims to explore the possibility of treating pulpitis with S14G-humanin. LPS-stimulated dental pulp cells (DPCs) were utilized to simulate an inflammatory state in the progression of pulpitis. We found the elevated expressions and production of interleukin- 6 (IL-6), tumor necrosis factor-α (TNF-α), macrophage chemoattractant protein-1 (MCP-1), matrix metalloproteinase-2 (MMP-2), and matrix metalloproteinase-9 (MMP-9), upregulated Pentraxin 3 (PTX3) and activated oxidative stress in LPS-treated DPCs were all reversed by treatment with 50 and 100 μM S14G-humanin. In addition, the LPS-induced elevated expression levels of toll-like receptor 4 (TLR4) and myeloid differentiation primary response 88 (Myd88), and activation of the IκBα/NF-κB signaling pathway in hDPCs were significantly repressed by treatment with S14G-humanin. Conclusively, we found that S14G-humanin protected LPS-treated hDPCs by inhibiting the TLR4/MyD88/NF-κB signaling pathway.

## Introduction

Pulpitis is a common oral cavity disease mainly characterized by severe and excruciating pain, which significantly impacts the health and life quality of patients [1,2]. Dental pulp tissue infection induced by Gram-negative bacteria and its metabolites is regarded as the main inducer of pulpitis. It has been recently reported that the immune reactions in the dental pulp tissues play vital roles in the development and processing of pulpitis [3,4]. Dental pulp tissue mainly consists of dental pulp cells (DPCs), which produce pro-inflammatory factors by expressing adhesion molecules and recognizing microorganisms and pathogen-associated molecular patterns (PAMPs) [5–7]. Several patterns have been concluded for the external stimulation on dental pulp tissues to initiate the immune reactions: immunoglobulins located in dentin tubules, odontoblasts, nerve polypeptides secreted by peripheral nerve cells and associated neurogenic inflammatory responses, lipopolysaccharide (LPS), immune cells such as macrophages and T cells, inflammatory factors, and chemokines [8]. Following the exploration of the pathological mechanism of pulpitis, it is widely reported that DPCs are involved in its pathogenesis by producing multiple types of pro-inflammatory factors. Hirao claimed that in streptococcus mutans-stimulated DPCs, the expression levels of toll-like receptors (TLRs) and nucleotide-binding oligomerization domain (NOD) proteins were found to be significantly elevated [9]. The secretion of multiple inflammatory factors, such as interleukin-1 (IL-1), transforming growth factor-β (TGF-β), and vascular endothelial growth factor (VEGF), in DPCs are facilitated by the stimulation of Gram-negative bacteria and their metabolites. These inflammatory factors subsequently recognize pattern recognition factors (PRPs), such as TLRs, to activate the immune system [10–12]. TLR4 is the first reported mammalian TLR, and LPS released by the Gram-negative bacteria has been proven to be the main ligand of TLR4, which regulates the inflammatory reaction in a MyD88- and TRIF- dependent manner [13]. TLR4 is widely reported to be highly expressed in DPCs during the development of pulpitis [14,15]. Under the stimulation of LPS, TLR4 is activated to pass signals through the MyD88 pathway, activating NF-κB to induce the secretion of inflammatory cytokines [16]. Therefore, inhibiting TLR4 signaling might be an effective method for the treatment of pulpitis.

Humanin is a linear peptide isolated from the occipital lobe of Alzheimer’s disease patients using the cDNA library method, and S14G-humanin is one of its derivatives. Approximately 1000-fold of neuroprotective property is reported on S14G-humanin compared to humanin [17]. S14G-humanin is proven to exert neuroprotective effects by suppressing oxidative stress, alleviating synaptic function, and reducing neuron apoptosis [18,19]. Recently, significant anti-inflammatory effects of S14G-humanin have been widely claimed [20]. Peng *et al*. reported that S14G-humanin inhibited the expression of pro-inflammatory cytokines, including TNF-α, IL-1β, IL-6, and MCP-1 in a murine stroke model. Meanwhile, they found that S14G-humanin treatment reduced the attachment of monocytes to bEnd.3 cells through modulating the NF-κB signaling pathway [21]. However, whether S14G-humanin exerts any protective effect in pulpitis is still unknown. In the current study, the beneficial effect of S14G-humanin in LPS-stimulated DPCs will be investigated to achieve the possibility of treating pulpitis with S14G-humanin.

## Materials and methods

### Isolation and cell culture of human DPCs (hDPCs)

This study was performed following the principles of the World Medical Association Declaration of Helsinki for medical research using human-derived cells. All experiments were carried out in accordance with the protocols approved by the Ethical Committee of Changzhi Medical college. The hDPCs were obtained from a healthy third molar isolated from an 18-year-old volunteer who was undergoing orthodontic treatment. In brief, pulp tissues were separated and rinsed using the phosphate buffered saline (PBS), followed by being digested using 2 mg/ml collagenase and 0.25% trypsin for half an hour. The suspension was then centrifugated, and the collected cells were resuspended in α-MEM (Invitrogen, New York, USA) supplemented with 10% fetal bovine serum (FBS), at 5% CO_2_ and 37°C conditions. The hDPCs were co-treated with LPS (1 μg/ml) (Sigma Aldrich, USA) with or without S14G-humanin (50, 100 μM) (GenScript, New Jersey, USA) for 6 hours.

### 3-(4, 5-dimethylthiazol-2-yl)-2, 5-diphenyl tetrazolium bromide (MTT) assay

Treated hDPCs were seeded in the 96-well plate to be incubated for 4 hours, followed by adding 20 μL 2 mg/mL MTT solution (Sigma Aldrich, USA) into each well. After 4 hours of incubation, the formazan crystal pellets dissolved in dimethyl sulfoxide (DMSO) were added, and the absorbance at 570 nm was detected using the microplate reader (Bio-Red, California, USA) [22].

### Real-time PCR assay

Cells were collected, and the total RNAs were extracted from cells using the TRIzol reagent (Invitrogen, California, USA), followed by transcribing the RNAs into cDNAs with a PrimeScript RT Reagent Kit (Takara, Tokyo, Japan). The PCR in the present study was conducted using the SYBR-Green dye (Takara, Tokyo, Japan) and a 7500 Real-Time PCR System (Invitrogen, California, USA). The relative expression level of target genes was determined by the 2^−ΔΔCt^ method after normalization with the glyceraldehyde-3-phosphate dehydrogenase (GAPDH) expression level. The following primers were used in this study:

TNF-α,F:5ʹ-ATTGGCAAATGGGAAAATGA −3ʹ, R:5ʹ-TTATGACCTCCTTTTGGT CTGA −3ʹ; IL-6, F:5′-AAAGAGGCACTGCCAGAAAA-3′, R:5′-ATCTGAGGTGCC CATGCTAC-3′; PTX3, F:5′-TTGGACAACGAAATAGACAATGGA-3′, R:5′-GTCG TCCGTGGCTTGCA-3′; TLR4, F: 5ʹ-CAGAGTTTCCTGCAATGGATCA-3ʹ, R: 5ʹ-G CTTATCTGAAGGTGTTGCACAT-3ʹ; MMP-2, F: 5ʹ-TTTCCATTCCGCTTCCAGG GCAC-3ʹ, R: 5ʹ-TCGCACACCACATCTTTCCGTCACT-3ʹ; MMP-9, F:5′-GCTAC GTGACCTATGACATCCTG-3′, R:5′ -AGAAACACTCCAACAAAAAACAAAG-3′; MCP-1,F:5′-CGCTCAGCCAGATGCAATCAATG-3′, R:5′-ATGGTCTTGAAGATC

ACAGCTTCTTTGG-3; MyD88, F:5ʹ-TCTCTGTTCTTGAACGTGCGGACA-3ʹ, R: 5ʹ-TTTGGCAATCCTCCTCAATGCTGG-3ʹ; GAPDH, F:5′-AGCCTCAAGATCAT CAGCAA −3′, R:5′- GTCATGAGTCCTTCCACGAT-3′.

### Enzyme-linked immunosorbent assay (ELISA)

The secretions of IL-6, TNF-α, MCP-1, MMP-2, and MMP-9 were measured using ELISA assay. In brief, the supernatant, together with the standards, were seeded on the 96-well plate and incubated at 37°C for 30 min, followed by removing the samples and 3 washes. After adding the conjugate reagents to be incubated for 1.5 h, the TMB solution was added into each well, followed by incubation for 15 min at 37°C. Lastly, the reaction was terminated by adding the stop solution, and the microplate reader (Bio-Red, California, USA) was used to measure the absorbance at 450 nm to calculate the concentrations.

### Western blot assay

Cells were lysed using the lysis buffer, and the total proteins were extracted, followed by quantification using the bicinchoninic acid (BCA) kit (ZIKER, Guangdong, China). After loading about 40 µg of proteins onto the 12% sodium dodecyl sulfate-polyacrylamide gel electrophoresis (SDS-PAGE), proteins were separated and transferred to the polyvinylidene fluoride (PVDF) membrane, and then incubated with 5% skim milk. Then the membrane was incubated with the primary antibody against PTX3 (1:1000, #sc-373,951, Santa Cruz Biotechnology, California, USA), TLR4 (1:1000, #sc-293,072, Santa Cruz Biotechnology, California, USA), Myd88 (1:1000, #sc-74,532, Santa Cruz Biotechnology, USA), p-IκBα (1:1000, #sc-8404, Santa Cruz Biotechnology, USA), IκBα (1:1000, #sc-52,900, Santa Cruz Biotechnology, USA), NF-κB p65 (1:1000, #sc-8008, Santa Cruz Biotechnology, USA), and β-tubulin (1:1000, #sc-166,729, Santa Cruz Biotechnology, USA), respectively, followed by 3 washes, and being incubated with the secondary antibody (1:1000, Santa Cruz Biotechnology, California, USA). Lastly, the bands were exposed to the ECL solution, and the images were quantified using the Image J software.

### Measurement of mitochondrial reactive oxygen species (ROS)

The Mito SOX Red staining assay was used to measure the mitochondrial ROS level. In brief, treated hPDCs were incubated with 5 μM MitoSox Red at 37°C for 10 min, followed by 3 washes. The images were taken with a fluorescence microscope (KEYENCE, Tokyo, Japan) [23].

### Determination of reduced glutathione (GSH)

The production of reduced GSH in the supernatant of treated hDPCs was detected using the commercial kit (Cayman, Ann Arbor, MI, USA) according to the method described previously [24].

### Luciferase activity of NF-κB promoter

Treated hDPCs were transfected with the pNF-κB Luc reporter plasmid (Beyotime, Shanghai, China) and the lipofectamine 3000 (Thermo Fisher, Massachusetts, USA), followed by being incubated for 48 hours. The reporter activity was determined utilizing a luciferase reporter kit (Biovision, Los Angeles, USA) using a synergy microplate reader (Bio-Red, California, USA).

## Statistical analysis

Data achieved from the present study was expressed with mean ± SD, which were analyzed using the GraphPad software by determining the difference between two groups with the Student t-test and the difference among groups using the ANOVA method, followed by Tukey’s post-hoc test. P < 0.05 was taken as a significant difference.

## Results

In this study, we used an LPS-challenged hDPCs model to examine the effects of S14G-humanin on bacterial infection-associated inflammatory response and damages. Our results demonstrate that S14G-humanin attenuated the expressions of IL-6, TNF-α, MCP-1, MMP-2, MMP-9, and PTX3. Importantly, treatment with S14G-humanin ameliorated LPS-induced oxidative stress in hDPCs. Also, we proved that these effects are mediated through blockage of the TLR4/MyD88/NF-κB signaling pathway.

### Effects of S14G-humanin on the cell viability of hDPCs

**Figure 1.**
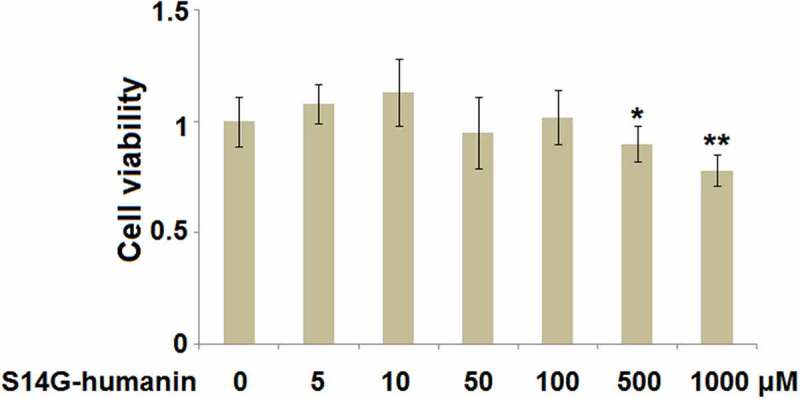
Effects of S14G-humanin in cell viability of human dental pulp cells (hDPCs). hDPCs were treated with S14G-humanin at the concentrations of 0, 5, 10, 50, 100, 500, 1000 μM for 24 hours. Cell viability was determined (*, **, P < 0.05, 0.01 vs. vehicle group)

To avoid cytotoxicity of S14G-humanin on hDPCs, cells were treated with S14G-humanin at the concentrations of 0, 5, 10, 50, 100, 500, 1000 μM for 24 hours followed by determining the cell viability. As shown in Figure 1, as the concentration of S14G-humanin increased from 0 to 100 μM, the cell viability was slightly changed. However, when the concentration of S14G-humanin exceeded 500 μM, the cell viability decreased significantly. Therefore, 50 and 100 μM were utilized as the incubation concentrations of S14G-humanin in hDPCs.

### S14G-humanin attenuated the excessive expression and production of IL-6, TNF-α, and MCP-1 induced by LPS in hDPCs

**Figure 2.**
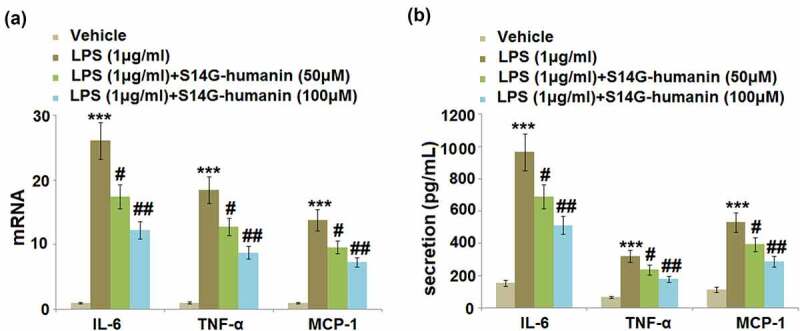
S14G-humanin attenuated the expression of IL-6, TNF-α, MCP-1 induced by LPS in hDPCs. The hDPCs were treated with LPS (1 μg/ml) with or without S14G-humanin (50, 100 μM) for 6 hours. (a). The mRNA Level of IL-6, TNF-α, and MCP-1; (b) The secretion levels of IL-6, TNF-α, and MCP-1 (***, P < 0.001 vs. vehicle group; #, ##, P < 0.05, 0.01 vs. LPS group)

Excessive production of inflammatory factors is an important symptom of pulpitis in dental pulp tissues isolated from patients. The hDPCs were treated with LPS (1 μg/ml) with or without S14G-humanin (50, 100 μM) for 6 hours, followed by detecting the secretion of pro-inflammatory factors in different groups. The gene expression levels of IL-1β, TNF-α, and MCP-1 (Figure 2(a)) were greatly promoted by LPS but then significantly repressed by treatment with 50 and 100 μM S14G-humanin. At the protein level (Figure 2(b)), the production of IL-6 was dramatically increased from 153.8 pg/mL to 966.5 pg/mL in the LPS group, then suppressed to 689.5 and 513.7 pg/mL by the incubation with 50 and 100 μM S14G-humanin, respectively. The secretions of TNF-α in the control, LPS, 50, and 100 μM S14G-humanin groups were 67.2, 321.6, 236.7, and178.9 pg/mL, respectively. In addition, compared to the control, the concentration of MCP-1 was significantly promoted from 113.7 pg/mL to 531.2 pg/mL by LPS, then greatly declined to 395.7 and 288.9 pg/mL by 50 and 100 μM S14G-humanin, respectively. We found that inflammatory reactions in LPS-treated hDPCs were dramatically ameliorated by S14G-humanin.

### S14G-humanin attenuated the expression of MMP-2 and MMP-9 induced by LPS in hDPCs

**Figure 3.**
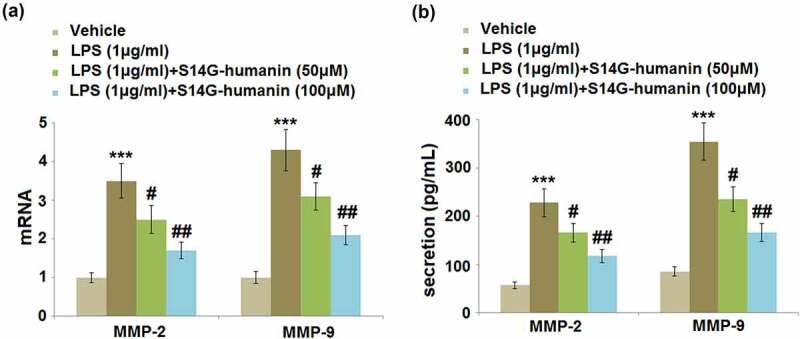
S14G-humanin attenuated the expression of MMP-2 and MMP-9 induced by LPS in hDPCs. (a)The mRNA Levels of MMP-2 and MMP-9; (b) The secretion levels of MMP-2 and MMP-9 were determined by ELISA (***, P < 0.001 vs. vehicle group; #, ##, P < 0.05, 0.01 vs. LPS group)

Matrix metalloproteinases (MMPs) are important inducers of inflammation in hDPCs during pulpitis. We further evaluated the gene and protein expression levels of MMP-2 and MMP-9 in different groups. As shown in Figure 3(a), compared to the control, the gene expression levels of MMP-2 and MMP-9 were significantly elevated by LPS but greatly inhibited by the introduction of 50 and 100 μM S14G-humanin. At the protein level (Figure 3(b)), the secretions of MMP-2 in the control, LPS, 50, and 100 μM S14G-humanin groups were 56.8, 228.1, 165.9, and 117.8 pg/mL, respectively. In addition, compared to the control, the concentration of MMP-9 was dramatically increased from 85.7 pg/mL to 354.6 pg/mL by the incubation with LPS, which was significantly repressed to 235.7 and 166.7 pg/mL in the 50 and 100 μM S14G-humanin group, respectively. These data reveal that the increased expression levels of MMPs in LPS-stimulated hDPCs were significantly reversed by S14G-humanin.

### S14G-humanin attenuated the elevated expression of Pentraxin 3 (PTX3) induced by LPS in hDPCs

**Figure 4.**
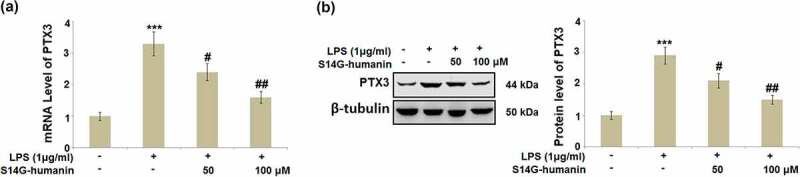
S14G-humanin attenuated the elevated expression of Pentraxin 3 (PTX3) induced by LPS in hDPCs. (a)The mRNA Level of PTX3; (b) Protein level of PTX3 (***, P < 0.001 vs. vehicle group; #, ##, P < 0.05, 0.01 vs. LPS group)

Pentraxin 3 is widely reported to be a regulator for the production of inflammatory factors, including in the development of pulpitis [25]. The expression level of PTX3 in different groups was subsequently measured. As shown in Figure 4, compared to the control, PTX3 was found to be significantly upregulated in LPS-stimulated hDPCs, then dramatically downregulated by treatment with 50 and 100 μM S14G-humanin, respectively.

### S14G-humanin ameliorated LPS-induced oxidative stress in hDPCs

**Figure 5.**
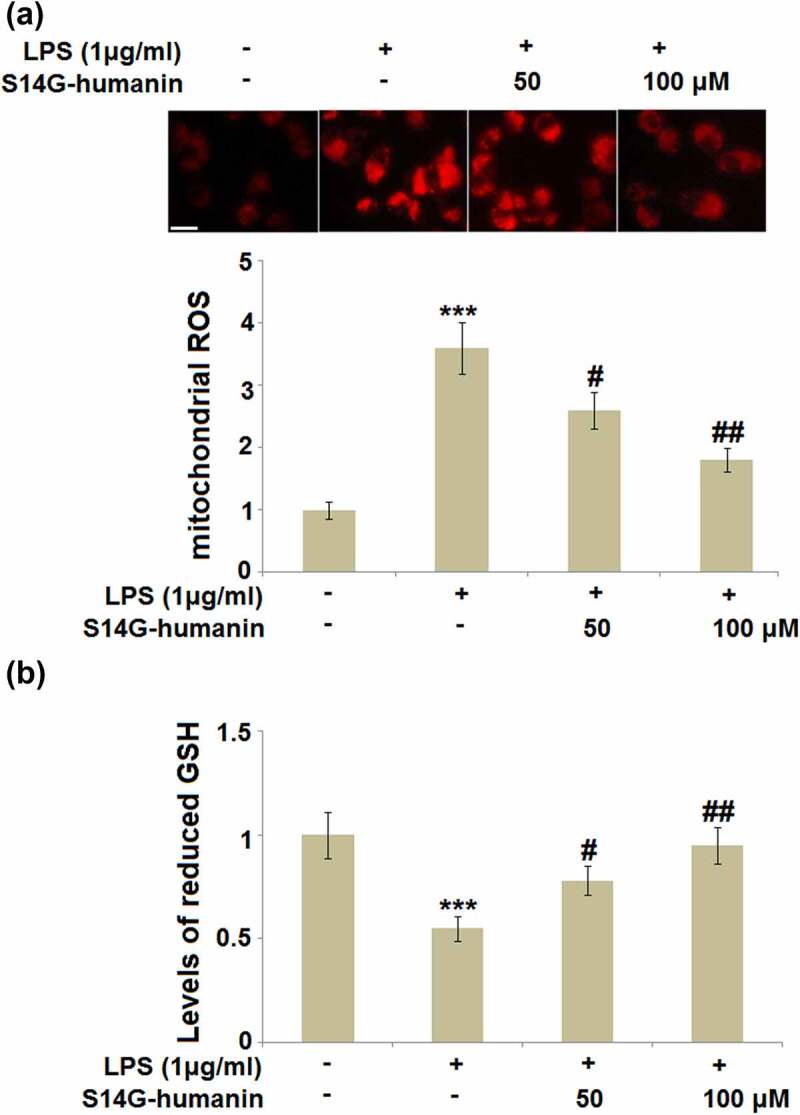
S14G-humanin ameliorated LPS-induced oxidative stress in hDPCs. (a). Levels of mitochondrial ROS; Scale bar, 100 μm; (b). Levels of reduced GSH (***, P < 0.001 vs. vehicle group; #, ##, P < 0.05, 0.01 vs. LPS group)

Inflammation is frequently accompanied by the activation of oxidative stress, which has been proven to be involved in the development of pulpitis [26]. The production of ROS is a classic marker of oxidative stress, and the level of reduced GSH is a reversing indicator for oxidative stress [27]. As shown in Figure 5, compared to the control, the production of ROS was significantly promoted, and the level of reduced GSH was significantly declined by the stimulation with LPS, both of which were reversed by treatment with 50 and 100 μM S14G-humanin, indicating its inhibitory effect on oxidative stress in LPS-stimulated hDPCs.

### S14G-humanin suppressed LPS-challenged activation of TLR4/Myd88 in hDPCs

**Figure 6.**
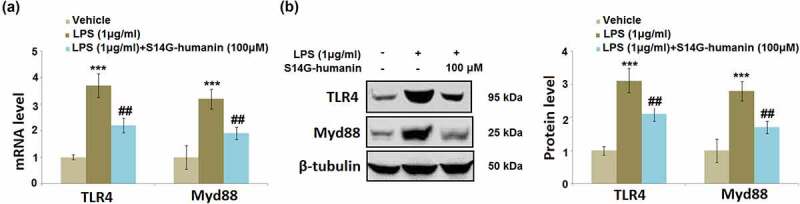
S14G-humanin reduced LPS-induced expression of TLR4/Myd88 in hDPCs. Cells were treated by LPS (1 μg/ml) with or without S14G-humanin (100 μM) for 6 hours. (a). mRNA of TLR4 and Myd88; (b). Protein levels of TLR4 and Myd88 (***, P < 0.001 vs. vehicle group; ##, P < 0.01 vs. LPS group)

The TLR4/Myd88 pathway is an important inflammatory pathway in the downstream of the interaction between LPS and TLR4 ^16^. Cells were treated with LPS (1 μg/ml) with or without S14G-humanin (100 μM) for 6 hours. As shown in Figure 6, compared to the control, the expression levels of TLR4 and Myd88 were significantly elevated by the stimulation with LPS, then greatly repressed by the introduction of 100 μM S14G-humanin, indicating a promising inhibitory effect of S14G-humanin on the TLR4/Myd88 pathway.

### S14G-humanin ameliorated LPS-induced activation of IκBα/NF-κB

**Figure 7.**
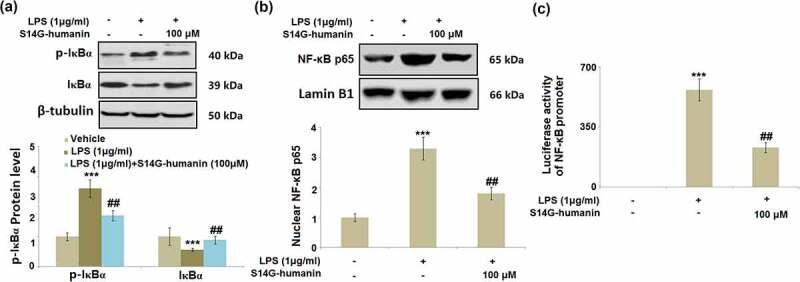
S14G-humanin ameliorated LPS-induced activation of IκBα/NF-κB. Cells were treated by LPS (1 μg/ml) with or without S14G-humanin (100 μM) for 6 hours. (a). Levels of p-IκBα and total IκBα; (b). Nuclear NF-κB p65; (c). Luciferase activity of NF-κB promoter (***, P < 0.001 vs. vehicle group; ##, P < 0.01 vs. LPS group)

NF-κB has been identified as an important transcriptional factor involved in the production of pro-inflammatory factors [28]. As shown in Figure 7(a), compared to the control, the expression level of p-IκBα was dramatically promoted, and the expression level of total IκBα suppressed in LPS-stimulated hDPCs, both of which were reversed by the introduction of S14G-humanin. In addition, the upregulated NF-κB p65 (Figure 7(b)) in the nuclei of cells in the LPS group was significantly downregulated in the 100 μM S14G-humanin group. Lastly, the activated luciferase activity of the NF-κB promoter in LPS-stimulated hDPCs was pronouncedly suppressed by S14G-humanin, indicating that the activated IκBα/NF-κB pathway in LPS-challenged hDPCs was dramatically alleviated by S14G-humanin.

### Overexpression of TLR4 abolished the protective effects of S14G-humanin against LPS- induced inflammatory response

**Figure 8.**
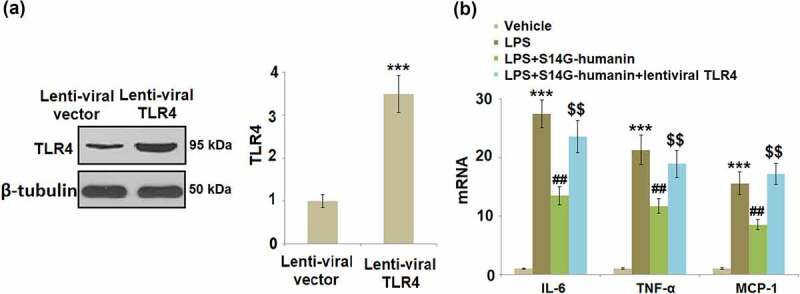
**Overexpression of TLR4 abolished the protective effects of S14G-humanin against LPS- induced inflammatory response**. Cells were transduced with lentiviral TLR4, followed by stimulation with LPS (1 μg/ml) with or without S14G-humanin (100 μM) for 6 hours. (a). Levels of TLR4; (b). mRNA of IL-6, TNF-α, and MCP-1 (***, P < 0.001 vs. vehicle group; ##, P < 0.01 vs. LPS group; $$, P < 0.01 vs. LPS+ S14G-humanin group)

To further confirm that the beneficial effects of S14G-humanin against LPS- induced inflammatory response are mediated by inhibiting the TLR4/IκBα/NF-κB signaling pathway, hDPCs were transduced with lentiviral TLR4. Successful overexpression of TLR4 is shown in Figure 8(a). Importantly, we found that the inhibitory effects of S14G-humanin against the LPS- induced production of IL-6, TNF-α, and MCP-1 were abolished by the overexpression of TLR4.

## Discussion

Several reports claimed the significance of severe inflammation in the development and progression of pulpitis [29,30]. Excessive production of pro-inflammatory factors has been widely observed in clinical [31] and animal experiments [32]. In the present study, LPS-treated hDPCs were utilized to simulate an inflammatory state in the progression of pulpitis, which was identified by the elevated secretion of pro-inflammatory factors. After S14G-humanin treatment, the severe inflammation in hDPCs was significantly alleviated, demonstrating the anti-inflammatory effect of S14G-humanin. PTX3 is a novel regulator of inflammation being paid high attention, involved in regulating inflammation in the progression of pulpitis [25]. In the present study, we found that the upregulated PTX3 in LPS-treated hDPCs was reversed by S14G-humanin, indicating that the anti-inflammatory effect of S14G-humanin might be related to its inhibitory effect on PTX3. In our future work, the interaction between S14G-humanin and PTX3 will be further investigated to explore the potential anti-inflammatory mechanism of S14G-humanin.

As a special connective tissue, the dental pulp is mainly composed of dental pulp cells and extracellular matrix (ECM). ECM in the dental pulp tissues is composed of biomacromolecules secreted by dental pulp cells or odontoblasts, which affect the morphology, migration, proliferation, and differentiation of dental pulp cells [33]. MMPs belong to the family of endopeptidase and are involved in the pathogenesis of various diseases by degrading ECM [34]. They have been identified to play an important role in the pathological mechanism of pulpitis [35]. In the present study, MMP-2 and MMP-9 were significantly upregulated in LPS-stimulated hDPCs and further repressed by treatment with S14G-humanin, indicating a potential protective effect of S14G-humanin on the impaired ECM during the progression of pulpitis.

Under external stimulation, the production of ROS are enhanced. When the excessive released ROS cannot be removed by the anti-oxidative system, oxidative stress is activated [36]. In the present study, we found that the increased mitochondrial ROS level and declined reduced GSH level were observed in LPS-treated hDPCs and dramatically reversed by S14G-humanin, indicating that the activation of oxidative stress in LPS-treated hDPCs was attenuated by S14G-humanin. In our future work, the effects of S14G-humanin on the mitochondrial function and the pathway regulating oxidative stress, such as the Nrf2 pathway, will be further investigated to figure out the impact of S14G-humanin on the oxidative injury.

TLR4 bridges with MyD88 through the TIRAP/Mal axis, and MyD88 binds to the IL-1 R associated kinase-4 (IRAK-4), further activating other IRAKs family members [37]. IRAKs subsequently separate from MyD88 to bind with TRAF6 and induce the formation of TAB2 and TAK1, contributing to the formation of IKK-α and IKK-β complex. As a consequence, the p-IκB, a natural inhibitor for NF-κB, is activated [38], triggering the transfer of NF-κB from the cytoplasm into the nucleus, which finally results in the gene expression of inflammatory factors [39]. The TLR4/MyD88/NF-κB signaling was dramatically initiated in LPS-stimulated hDPCs and further repressed by treatment with S14G-humanin, indicating that S14G-humanin exerted anti-inflammatory properties by inhibiting the TLR4/MyD88/NF-κB pathway.

## Conclusion

In conclusion, our data show that S14G-humanin inhibited inflammatory response in LPS-treated hDPCs by reducing the expression of pro-inflammatory cytokines and MMPs. Furthermore, S14G-humanin attenuated oxidative stress by suppressing ROS generation and increasing the levels of reduced GSH. Importantly, we found that the protective effects of S14G-humanin are mediated by the TLR4/MyD88/NF-κB signaling pathway. These findings suggest that S14G-humanin may have the potential to treat Pulpitis.
